# Supplementary material for: Cancer LncRNA Census 2 (CLC2): an enhanced resource reveals clinical features of cancer lncRNAs
Source: NAR Cancer. 2021 Apr 14;3(2):zcab013. doi: 10.1093/narcan/zcab013 (PMC8210278; doi:10.1093/narcan/zcab013)
Supplement: zcab013_Supplemental_Files [file zcab013_supplemental_files.zip › Supp_Data_NARcancer_revised.docx]

**Supplementary Data**

**Supplementary Table 1:** Excel table with all CLC2 and cancer type and evidence level

**Supplementary Table 2:** Excel table with all CLC2 ENSG with cancer functionality

**Supplementary Figures**

**SUPP. Figure 1:** Insertion analysis

**A)** All insertion sizes after LiftOver compared to GENCODE v28 gene length. Number of input CIS elements in mouse compared to hCIS elements in human after LiftOver. **B)** Assign genes to GENCODE v28 genes and gene families. **C)** CCGD reported genes (dark) and CLIO-TIM reported genes (fade) for each gene class. **D)** Genes with insertion categorized in gene types. Statistical significance calculated by one-sided Fisher’s test.  **E)** Assign intergenic regions to MiTranscriptome IDs and compare to shuffled hCIS overlayed with MiTranscriptome IDs. Example of one insertion site with MiTranscriptome ID.

**SUPP. Figure 2:** *LINC00570* insertion candidate characteristics.

**A)** ENCODE expression data of *LINC00570* in HeLa (blue)  and HCT116 cells (black). **B)** Cell proliferation of HeLa cells treated with ASO negative control and ASO 3 at Day 1, 2 and 3. **C)**

Right side indicates LINC00570 qPCR product at 95bp in size. Left side depicts primer details for LINC00570 and the sequenced qPCR product. **D)** Expression of *ROCK2* in relation to *GAPDH* in response to inhibition by CRISPRi (left) or ASOs (right) in n=4 (or else indicated) biological replicates.

**SUPP. Figure 3:** Comparison of first CLC and CLC2.

**A)** CLC2 genes are detected in 33 cancer types and compared to 29 in the first CLC. CLC reported n=122 literature lncRNAs whereas CLC2 comprises 492 genes from 3 different analysis. **B)** Comparing evidence levels of genes from the initial CLC with the CLC2.

**SUPP. Figure 4:** CLC2 expression and gene length bias.

**A)** CLC2 genes (turquoise) are higher expressed than nonCLC genes (grey), same for CGC genes (red) compared to nonCGC genes (orange). Expression-matched CLC2 (blue) and CGC (yellow) were generated and match the expression of the CLC2 and CGC, respectively. **B)** CLC2 genes (turquoise) with increased exon and whole gene body length when compared to expression-matched (blue) and all other lncRNAs (grey). **C)** CLC2 genes are enriched for transcripts with coding potential compared to nonCLCmatched and nonCLC genes. Statistical significance calculated by one-sided Fisher’s test.

**SUPP. Figure 5:** CLC2 gene characteristics.

**A)** CLC2 genes are enriched for ⅔ of the analyzed repeat element families when compared to expression-matched nonCLC genes. Statistical significance calculated by hypergeometric test (highly significant ****=<0.0001). **B)** CGC and CLC2 genes are enriched for small RNAs compared to expression-matched nonCGC and nonCLC, respectively. In the bar graph we report the fraction of genes of each dataset with (dark color) or without (light color) small RNA encoded in the genomic region. Statistical significance calculated by one-sided Fisher’s test. **C)** Genomic classification of CLC2, expression-matched nonCLC and nonCLC genes. Statistical significance calculated by two-sided Fisher’s test (*=<0.05). **D)** Genomic classification of CLC2, corresponding to CGC and nonCGC genes. Statistical significance calculated by Chi-square with Yates' correction test (highly significant ****=<0.0001).

**SUPP. Figure 6:** TCGA cancer types for differential expression analysis.

**A)** CLC2 cancer types corresponding to TCGA cancer types. **B)** Samples for each TCGA cancer type analyzed for differential expression analysis.

**SUPP. Figure 7:** Cancer characteristics for all analyzed gene types.

**A)** Differentially expressed genes enriched in cancer-associated gene families (CGC and CLC2). Statistical significance calculated by one-sided Fisher’s test.  **B)** Exonic cancer SNPs enriched in cancer-associated gene families (CGC and CLC2). Statistical significance calculated by one-sided Fisher’s test.  **C)** Survival analysis comparing most significant p-value for each lncRNA in the CLC2 compared to expression-matched lncRNAs. Statistical significance calculated by ks-test.  **D)** CLC2 exons exhibiting a cancer SNP are less conserved than CLC2 exons in general; the conservation scores are comparable to expression matched nonCLC exon conservation. Statistical significance calculated by one-sided Fisher’s test.
