# Supplementary material for: Cancer LncRNA Census 2 (CLC2): an enhanced resource reveals clinical features of cancer lncRNAs
Source: NAR Cancer. 2021 Apr 14;3(2):zcab013. doi: 10.1093/narcan/zcab013 (PMC8210278; doi:10.1093/narcan/zcab013)
Supplement: zcab013_Supplemental_Files [file zcab013_supplemental_files.zip › Supp Figures.zip!/FiguresCLC2_NARcancer_revised_SUPP_Fig6.pdf]

A

CLC2 cancer type	TCGA cancer type
bile duct	CHOL
blood	DLBC
blood	LAML
breast	BRCA
cervical	CESC
colon	COAD
endometrial	UCEC
endometrial	UCS
esophageal	ESCA
head and neck	HNSC
kidney	KIRP
kidney	KIRC
kidney	KICH
liver	LIHC
lung	LUAD
lung	LUSC
neuroepithelial	LGG
neuroepithelial	PCPG
neuroepithelial	GBM
ovarian	OV
pancreas	PAAD
prostate	PRAD
skin	UVM
skin	SKCM
stomach	STAD
testis	TGCT
thyroid	THCA
urothelial	BLCA
sarcoma	SARC

B

TCGA cancer type	tumor (01)	healthy (11)	total samples
TCGA-BLCA	19	19	**38**
TCGA-BRCA	112	112	**224**
TCGA-CESC	3	3	**6**
TCGA-CHOL	9	9	**18**
TCGA-COAD	41	41	**82**
TCGA-ESCA	8	8	**16**
TCGA-HNSC	43	43	**86**
TCGA-KICH	23	23	**46**
TCGA-KIRC	72	72	**144**
TCGA-KIRP	31	31	**62**
TCGA-LIHC	50	50	**100**
TCGA-LUAD	57	57	**114**
TCGA-LUSC	49	49	**98**
TCGA-PAAD	4	4	**8**
TCGA-PCPG	3	3	**6**
TCGA-PRAD	52	52	**104**
TCGA-SARC	2	2	**4**
TCGA-STAD	27	27	**54**
TCGA-THCA	58	58	**116**
TCGA-UCEC	23	23	**46**
**TOTAL**			**1372**

SUPP. Figure 6
