# Supplementary material for: Cancer LncRNA Census 2 (CLC2): an enhanced resource reveals clinical features of cancer lncRNAs
Source: NAR Cancer. 2021 Apr 14;3(2):zcab013. doi: 10.1093/narcan/zcab013 (PMC8210278; doi:10.1093/narcan/zcab013)
Supplement: zcab013_Supplemental_Files [file zcab013_supplemental_files.zip › Supp Figures.zip!/FiguresCLC2_NARcancer_revised_SUPP_Fig3.pdf]

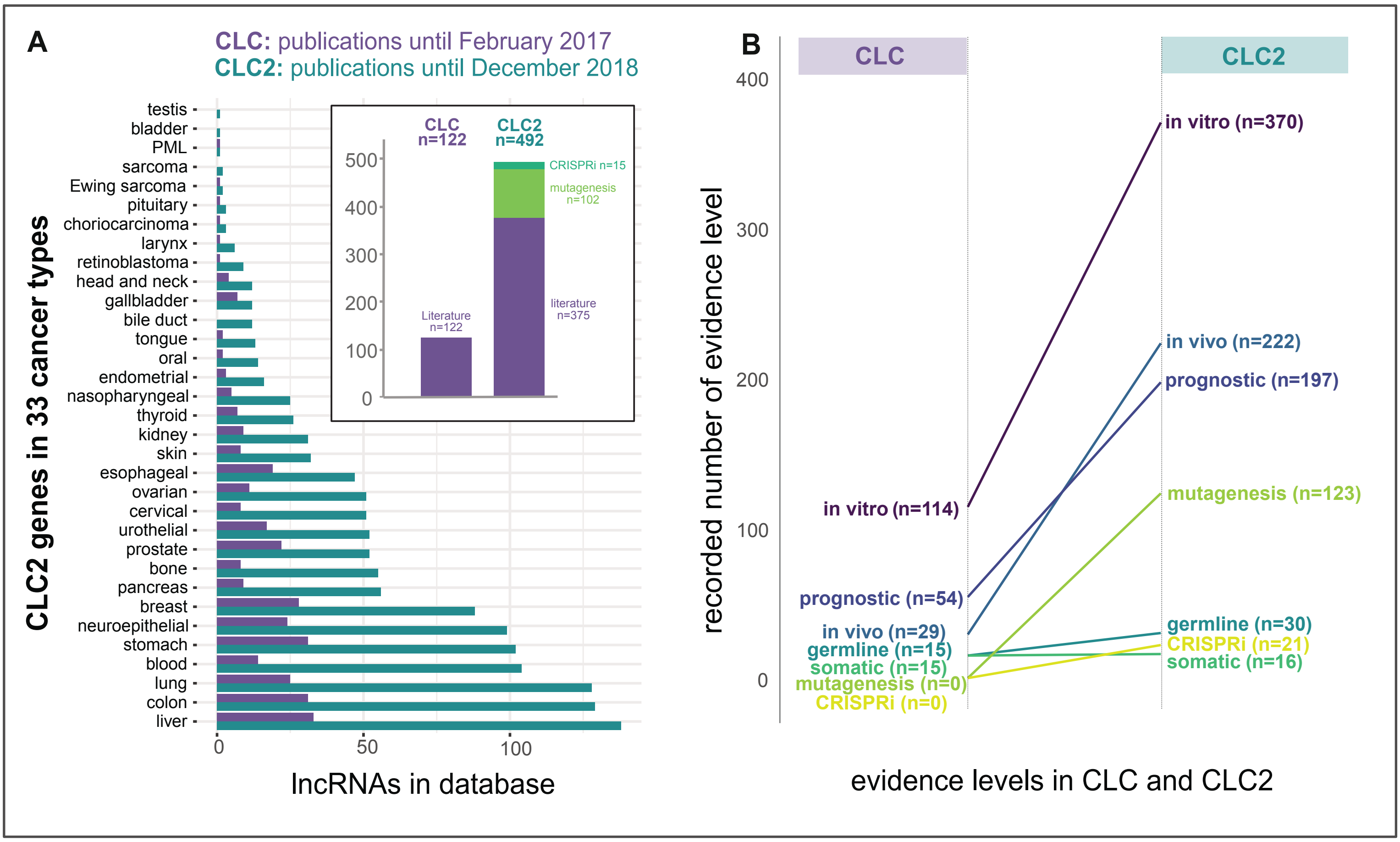

SUPP. Figure 3
